# Editorial: Biological and Robotic Inter-Limb Coordination

**DOI:** 10.3389/frobt.2022.875493

**Published:** 2022-03-22

**Authors:** Dai Owaki, Poramate Manoonpong, Amir Ayali

**Affiliations:** ^1^ Department of Robotics, Graduate School of Engineering, Tohoku University, Sendai, Japan; ^2^ Embodied AI and Neurorobotics Lab., SDU Biorobotics, The Mærsk Mc-Kinney Møller Institute, University of Southern Denmark, Odense, Denmark; ^3^ Bio-inspired Robotics and Neural Engineering Lab., School of Information Science and Technology, Vidyasirimedhi Institute of Science and Technology, Rayong, Thailand; ^4^ School of Zoology, Faculty of Life Sciences, and Sagol School of Neuroscience, Tel Aviv University, Tel Aviv, Israel

**Keywords:** locomotion, legged animals, embodiment, bio-inspired robotics, robotics-inspired biology

## 1 Introduction

Animals on Earth have evolved to counteract the effect of gravity, negotiate terrestrial ground, and locomote efficiently for predation and survival. Locomotion is thus one of the fundamental functions of life. Through many cycles of evolutionary selection, both vertebrates and invertebrates have acquired sophisticated locomotor skills, exhibiting resilient and flexible locomotion in response to changes in body morphology, environment, and context, by coordinating leg movements, i.e., inter-limb coordination. Thus, understanding the inter-limb coordination mechanism is both essential for understanding the locomotive mechanism in legged animals and useful for establishing design principles for legged robots that can reproduce flexible and efficient locomotion resembling that exhibited in animals.

Understanding the principles of legged locomotion is a goal shared among biologists and robotics engineers, who have struggled to build multi-legged robots able to exhibit adaptive locomotion via inter-limb coordination. Although it is now possible to create a high-performance architecture, e.g., CPU/GPU, to control the movement of a robot, robots are still not able to carry out more than a small fraction of the complex and adaptive behaviors found in animals. Given the limited number of neurons that comprise a nervous system (insects for example possess only approximately 10^5^ to 10^6^ neurons in their nervous system) we must consider the potential role of not only intrinsic neural circuits in adaptations to dealing with unpredictable situations, but also that of the sensory feedback mechanisms that reflect body properties and physical interactions with the environment. Understanding the mechanisms that underlie adaptive locomotion contribute not only to biology but also to the field of robotics, by facilitating the design of durable and resilient legged robots capable of adapting to unpredictable and changing situations, much like animals.

**FIGURE 1:**
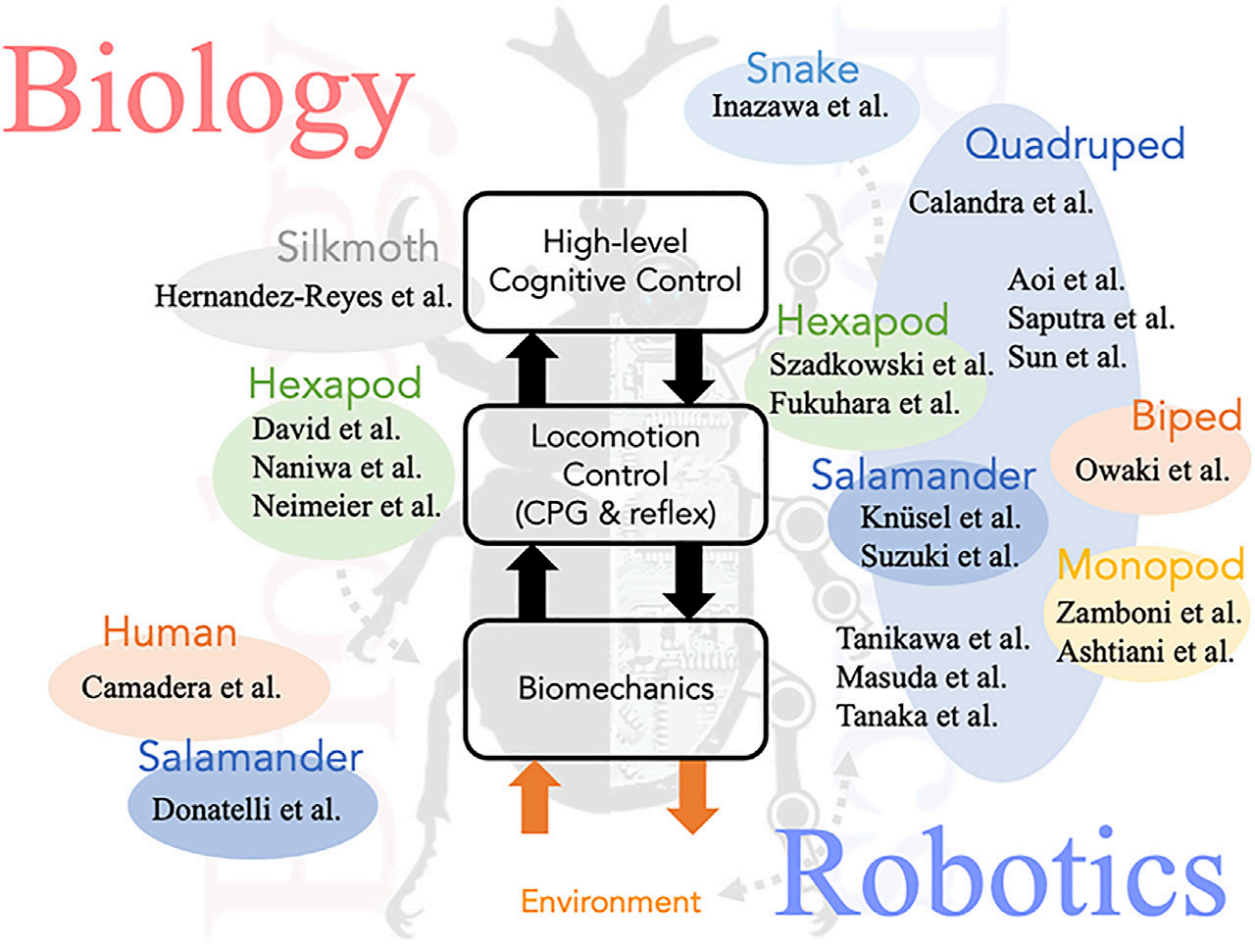
Overview of this Research Topic: “Biological and Robotics Inter-limb Coordination”.

Thus, the goal of this Research Topic is to consolidate topics related to “Biological and Robotic Inter-limb Coordination”, in order to encourage the acceleration of collaborative approaches between the fields of biology and robotics. The topic contains 22 articles, addressing biological and robotic inter-limb coordination mechanisms in different robotic and animal systems (Figure 1), as well as the translation of results to real-world applications, such as electromyographic (EMG)-based limb prostheses control.

## 2 Biological Inter-Limb Coordination

A major feature of animal locomotion is that of its flexibility or adaptability, i.e. the capacity of the locomoting animal to maintain robust and consistent movement through a variable and constantly changing environment. This outstanding ability, perfected by a long evolutionary history, is manifested at all levels of control and execution of locomotion: from the central and local neuronal circuits controlling and generating locomotion-related motor patterns, through the complex interactions of the central nervous system with ongoing as well as transient sensory signals, via the interactions of the central and sensory system with the muscular system responsible for executing movement of the body parts (legs, tail, and trunk), and last but not least, the dynamic interaction of these body parts with the physical environment (Figure 1, Center). A number of contributions to this special issue have addressed the distinctive plasticity of animal locomotion, focusing on aspects of locomotion and inter-limb coordination related to the different levels noted above (Figure 1, Left).

Starting with the central circuits, David and Ayali (2021) present a detailed investigation of the locomotion central pattern generating (CPG) neuronal networks and their underlying connectivity scheme in the cockroach, an established model in the study of locomotion control. They discuss the role of rhythmic properties of the endogenous local (segmental) CPGs vs inter-circuit coupling in the production of the functional, adaptable motor output during locomotion in the behaving animal. Yet another model insect, the cricket, is utilized by Naniwa and Aonuma (2021) in order to present the instrumental role of descending and ascending inputs into the thoracic motor control center (the CPGs controlling leg movements) in maintaining the walking pattern. They demonstrate that descending signals from the head ganglia play an inhibitory role in initiating leg movements; and that both the descending and ascending signals from the abdominal nervous system are important in initiating and coordinating the walking gait patterns.

Inter-limb coordination is directly investigated by Niemeier et al. (2021) by way of controlled lesions of thoracic connectives in one of the leading models in the study of leg coordination - the stick insect. The importance of neural information transfer among the legs is nicely demonstrated. Furthermore, the findings show that spatial and temporal coordination of leg movements are obtained independently, with the former rather than the latter being affected by the experimental manipulations. Overall this offers yet another example of the importance of proprioceptive feedback in the generation of a coordinated gait.

Insects search for and find the source of a desired odor as a basic locomotion behavior, such as when searching for food or a mate. This behavior offers an interesting research topic in regard to motor-cognitive function: how is such successful locomotion achieved under conditions of turbulent odor plumes, utilizing the insects’ small number of neurons. Hernandez-Reyes et al. (2021) measured the behavior of moths using a virtual reality system that presents accurate and reproducible odor stimuli by using blue light and optogenetic moths. Their results demonstrate that behavioral variations have a higher probability of obtaining more information than “programmed behaviors” (i.e., reactive, exploitative behaviors), suggesting that silk moths incorporate some stochasticity into their behavior in order to balance the exploration and exploitation of the acquired information.

Moving from invertebrate to vertebrate models, Donatelli et al. (2021) utilize the unique model of the bluespot salamander to demonstrate the robustness of locomotion patterns. Animals adjust their gait to a changing environment. In addition to adjusting to heterogeneities in their environment, animals can adjust their locomotion to contend with damage to appendages (tail or legs). This is ubiquitous in salamanders, which have the potential to regenerate missing limbs, tails, and even parts of the spinal cord in some species. As the authors suggest, understanding the changes that take place in locomotion kinematics as a lost limb regrows, may provide important insights to assist roboticists working on terrestrial as well as amphibious locomotion.

As noted above, one level of control, allowing consistency and also adaptability in inter-limb coordination; is the muscular system. Camardella et al. (2021) visit the cutting edge technique of myo-control: a type of brain-machine interface in which recorded electromyographic (EMG) signals are utilized in a computed control signal that drives robots or machines (e.g., limb prostheses control). For such applications, there is much interest in characterizing the minimum muscle set, termed an optimal set, that preserves performance and demonstrates a high consistency of motor activity. Such an optimal set is described as considering the best trade-off in terms of myo-control performance and muscle set size.

## 3 Robotic Inter-Limb Cooridination

To date, robotics research has made substantial progress in reproducing (adaptive) inter-limb coordination inspired by animal locomotion. The contributions to this special issue present various methods of control (including central and local (neural) control with sensory feedback) that have been investigated and developed, as well as validated on different animal-like robots (Figure 1, Right).

Starting with a snake-like robot, Inazawa et al. (2021) proposed a unified model-based method for designing the motion of a robot to deal with complicated pipe structures. The central control with a model considering slippage between robot and pipe coordinates the connected pitch-axis and yaw-axis joints of the robot body. It enables the robot to perform various maneuvers to deal with multiple pipe structures and obstacles such as junctions, bends, changes in pipe diameter, shears, and blockages.

Rather than employing system models for inter-limb coordination and locomotion generation, Owaki et al. (2021) proposed a model-free method for simulated bipedal robot locomotion. Their method is based on the Tegotae concept, which describes how well a perceived reaction based on sensory feedback matches the expectation (i.e., an intended motor command). The control method’s implementation makes use of vertical and horizontal ground reaction forces (GRF), as well as decentralized local control circuits, to allow the robot to walk on both flat and uneven terrains while adapting to environmental changes.

In addition to the snake and biped robots, several studies in this special issue employs quadruped robots as their experimental platforms in order to investigate and develop control mechanisms for adaptive inter-limb coordination. Sun et al. (2021) employed a simulated quadruped robot to investigate two classical adaptive inter-limb coordination mechanisms: continuous phase modulation (also known as Tegotae) and phase resetting. These mechanisms use decoupled neural central pattern generators (CPGs) or local neural control circuits with sensory feedback, such as GRFs, to generate self-organized robot locomotion. Theses authors compared the characteristics of the two mechanisms by observing the CPG phase convergence processes at different control parameter values. They also investigate the robustness of the mechanisms under various unexpected conditions, such as noisy feedback, leg motor damage, and carrying a load, in a simulated quadruped robot. From their findings, they suggest a strategy for the appropriate selection of adaptive inter-limb coordination mechanisms under different conditions and for the optimal setting of the control parameter values in order to enhance the control performance. Aoi et al. (2021) demonstrated the use of local CPG-based control with phase resetting as well as slow and fast adaptation mechanisms for quadrupedal locomotion on a split-belt treadmill. For the implementation, while the CPG control, modulated by phase resetting based on touch sensor signals (i.e., discrete GRFs) and desired (predicted) touchdown timing, forms adaptive inter-limb coordination, fast adaptation induces asymmetric inter-limb coordination following a change of the treadmill speed condition and slow adaptation slowly reduces or balances the asymmetry following fast adaptation. This leads to stable quadrupedal split-belt treadmill walking. Saputra et al. (2021) present central CPG-based control with multiple sensory feedback provided by exteroceptors (i.e., quad-composite time-of-flight and dual-laser range finder sensors), for detecting the surroundings and interoceptors (i.e., force and touch sensors and an inertial measurement unit (IMU)). Using this control approach, they are able to generate versatile locomotion and short-term adaptation for a cat-like quadruped robot. The robot can, consequently, walk on natural terrain, walk with a leg malfunction, avoid a sudden obstacle, and climb a vertical ladder.

Robust robot state estimation and sensory event mistiming detection are important issues for adaptive inter-limb coordination. Accordingly, Calandra et al. (2021) proposed a data-driven method using reservoir computing for translating local proprioceptive feedback, acquired at the leg joints of a simulated quadruped robot, into global exteroceptive information, which include both GRFs at the level of the different legs and information about the type of terrain traversed by the robot. This mechanism enables the robot to effectively estimate its walking state (i.e., estimating the GRFs from joint torques) and classify terrains for adaptive locomotion. Szadkowski et al. (2021) proposed a novel self-supervised method based on dynamic Hebbian-like rules for learning sensory event mistiming detection during robot walking. The sensory mistiming detector is integrated into central CPG-based control. Consequently, the CPG-based control engages with inter-limb coordination for gait generation while the detector engages with adaptive intra-limb coordination by triggering the elevator reflex, used to avoid an obstacle, and the search reflex, used to grasp at a missed foothold. This control method enables a hexapod robot to negotiate an unstructured and slippery subterranean environment. As well demonstrated by the biological studies in Section 1, yet far from fully resolved, insects excel in highly variable limb coordination patterns. Fukuhara et al. (2022) address this gap by proposing a simple mathematical model for the mechanism of variable inter-limb coordination in insect locomotion. Their model, largely based on active load sensing, was tested in simulation experiments and shown to entitle a hexapod robot with a range of typical gait patterns and improved adaptability in different locomotion speeds.

The salamander constitutes a model animal for focusing on the following two issues, representing an evolutionary process of moving from water to land: 1) versatile behavior generation against a changing environment, based on CPGs coordinated by both descending signals and sensory feedback; and 2) body-limb coordination, i.e., coordination between undulatory movements of the body and leg movements based on the salamander’s characteristic morphology. Using a robot, Knüsel et al. (2020) were able to reproduce the five motor behaviors observed in salamanders: swimming, struggling, forward underwater stepping, and forward and backward terrestrial stepping. A mathematical model is presented that allows the robot to switch between various motor patterns using a neural circuit with descending brain signals and proprioceptive feedback as input. The results suggest that a single flexible neural circuit contributes to the generation of various animal behaviors when modulated by descending drive and sensory feedback. When walking on level ground, the salamander coordinates not only its legs but also other body parts such as the trunk, head, and tail, i.e., body-limb coordination, to generate the standing and traveling waves of lateral bending depending on the walking speed and stride length. Suzuki et al. (2021) showed that a CPG-based controller with four feedback rules, limb-to-limb, limb-to-body, body-to-limb, and body-to-body, without assuming any inter-leg coupling is able to reproduce various walking patterns, suggesting that sensory feedback plays a crucial role in flexible body-limb coordination during sprawling quadruped locomotion.

Quadruped robots possess the minimum number of legs required for postural stability; hence, it is also a useful platform to discuss the effects of a lower level of control and of biomechanics, e.g., spinal reflexes or body softness, due to the high postural stability and relative simplicity of leg coordination control. Tanikawa et al. (2021) focused on spinal reflex, which is essential for quadruped walking, and experimentally verified the reflex mechanism using a robotic platform that mimics legs with high back-drivability and Hill-type muscle properties. Their findings suggest that the basic structure of the reflex circuit is that of the reciprocal coupling between extensor muscles via excitatory neural pathways, followed by the prolongation of the stance phase caused by the reciprocal excitatory reflex contributing greatly to the generation of a steady gait. Masuda et al. (2021) showed the feasibility of generating various quadruped gaits using only actuators and body dynamics. Although the developed robot has no sensors or microprocessors, its motors were able to autonomously adjust the phase according to the leg dynamics and its locomotion eventually converges to a stable gait pattern. Furthermore, by increasing the input voltage to the motors, the robot is able to reproduce pacing, bounding, rotary galloping, and half-bound-like lateral galloping. Tanaka et al. (2021) developed a quadruped robot driven by McKibben pneumatic artificial muscles and verified its turning motion. In particular, the experiments demonstrate that the softness of legs leads to adaptive changes in inter-leg coordination and enables the robot to turn dynamically, merely by changing the phase difference between the left and right hind legs. Their results suggest that a soft body can simplify the design of the controller for leg coordination in locomotion even for complex tasks.

Hopping motion offers an effective test-bed for theoretical approaches and systematic verification of energy optimization. The simple mechanical structure and constrained one-dimensional vertical motion allows simulation and robotic platform to uncover the contributing mechanisms and control schemes. Zamboni et al. (2021) investigated optimal energy efficiency of Tegotae control based on proprioceptive feedback previously used in bipedal, quadrupedal, and hexapod robot locomotion in the context of embodiment. For this purpose, simple one- and two-legged mechanical hopping robot simulation were conducted. Their results suggested that the Tegotae-based approach combined with a reflex-like actuation generate optimal energy-efficient motion as well as environmental adaptability and gait transitions. Discrete impact with the ground is a major factor of instability during legged locomotion due to their unknown timing and impact magnitude. Ashtiani et al. (2021) examined the effect of the combination of passive and active compliance on leg control during the landing event. Simulation and experiment with a single leg robot, followed by simulation with a quadruped robot were conducted. Their results showed that hybrid passive/active control was robust against feedback delays, comparable to the sensory-motor delays of neuromuscular systems in animals.

## 4 Concluding Remarks

In this research topic, we have brought together studies that provide an overview of recent developments in biological and robotic inter-limb coordination. Since inter-limb coordination constitutes the fundamental basis of motion control, the studies covered range from high-level cognitive functions to CPGs and spinal reflexes, as well as biomechanics and interaction with the environment. In terms of animal species, the studies in biological inter-leg coordination incorporate insects, salamanders, and human muscle control. The robotic inter-leg coordination studies, center on quadrupedal robots that display posture stability and controllability, and include monopod, biped, salamander, hexapod, and even snake locomotion. A birds-eye-view of the overall research topic reveals that “modeling,” including abstract mathematical description and physical implementation, is a key approach for discussing and understanding inter-leg coordination mechanisms in a unified manner encompassing biology and engineering. In addition, recent pioneering technologies, such as animal cyborgs that externally control the behavioral output of animals, and Virtual Reality (VR) or Augmented Reality (AR) systems that externally manipulate the sensory input to animals, are expected to lead to further understanding of the leg coordination mechanisms in animals. We hope that this research topic devoted to biological and robotic inter-limb coordination will help researchers to enter novel research areas related to leg coordination and that novel research results will ensue, based on the further integration of biology and robotics.
